# Müller cells and retinal angiogenesis: critical regulators in health and disease

**DOI:** 10.3389/fncel.2024.1513686

**Published:** 2024-12-10

**Authors:** Alan E. Medina-Arellano, Jesús Silvestre Albert-Garay, Tania Medina-Sánchez, Karla Hernández Fonseca, Matilde Ruiz-Cruz, Lenin Ochoa-de la Paz

**Affiliations:** ^1^Laboratorio de Neurobiología Molecular y Celular de la Glía, Facultad de Medicina, Departamento de Bioquímica, UNAM, Mexico City, Mexico; ^2^Unidad de Investigación APEC-UNAM, Asociación para Evitar la Ceguera en México I.A.P., Mexico City, Mexico; ^3^Programa de Doctorado en Ciencias Biomédicas, UNAM, Mexico City, Mexico; ^4^Laboratorio de Neuroquímica, Subdirección de Investigaciones Clínicas, Instituto Nacional de Psiquiatría “Ramón de la Fuente Muñiz”, Mexico City, Mexico

**Keywords:** Müller glial cells, angiogenesis, retina, secretome, cytokines

## Abstract

Müller cells are the most abundant glial cells in the mammalian retina. Their morphology and metabolism enable them to be in close contact and interact biochemically and physically with almost all retinal cell types, including neurons, pericytes, endothelial cells, and other glial cells, influencing their physiology by releasing bioactive molecules. Studies indicate that Müller glial cells are the primary source of angiogenic growth factor secretion in the neuroretina. Because of this, over the past decade, it has been postulated that Müller glial cells play a significant role in maintaining retinal vascular homeostasis, with potential implications in vasoproliferative retinopathies. This review aims to summarize the current understanding of the mechanisms by which Müller glial cells influence retinal angiogenesis in health and disease, with a particular emphasis on three of the retinopathies with the most significant impact on visual health worldwide: diabetic retinopathy, retinopathy of prematurity, and age-related macular degeneration.

## 1 Introduction

The vascular system is essential for delivering oxygen, nutrients, and metabolic precursors, transporting hormones and growth factors, and removing metabolic products (Burns et al., 2021). Angiogenesis, the formation of new blood vessels from existing ones (Pandya et al., 2006), determines the development of the normal retina and the loss of vision resulting from a group of diseases called vasoproliferative retinopathies, which include diabetic retinopathy (DR), retinopathy of prematurity (ROP), and age-related macular degeneration (AMD). Müller glial cells (MGC), the most abundant glial cell type in the retina, have emerged as critical regulators of angiogenesis in the retina. In response to various angiogenic stimuli, MGCs produce and secrete different growth factors and cytokines that ultimately regulate endothelial cells, pericytes, and other retinal cells to promote or inhibit angiogenesis (Coughlin et al., 2017; Subirada et al., 2018). Understanding the molecular mechanisms underlying MGC-mediated angiogenesis in the retina should contribute to developing therapeutic strategies for treating vasoproliferative retinopathies.

**Figure 1:**
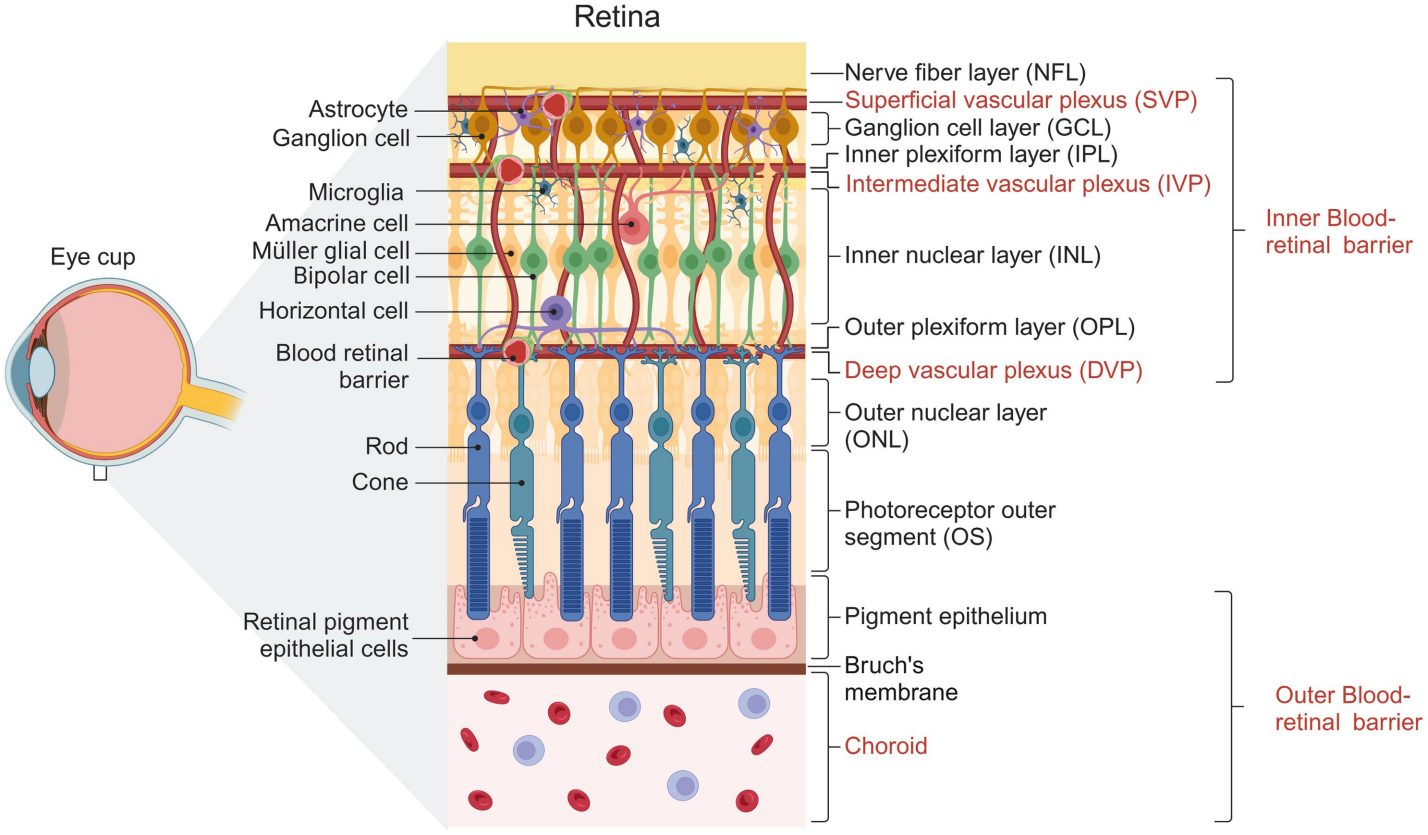
Representative diagram showing the structure of the human retina. Cells forming the different layers of the retina are shown. The three vascular plexuses supplying the inner retina and the choroid vessels supplying the outer retina are indicated. Created with BioRender.com

Vision is a complex physiological process that depends on the homeostasis of the retina. This tissue has unique functional and structural properties that allow it to detect and convert photons into chemical and electrical signals that are subsequently processed in the visual cortex. Structurally, the retina is divided into the retinal pigment epithelium (RPE) and the neural retina (NR). The NR is stratified into three nuclear and two plexiform layers (Kolb et al., 2018; Sadda et al., 2017), summarized in Figure 1. On the NR, glial cells regulate different processes necessary for the retina to function correctly (Reichenbach and Bringmann, 2020). Glial cells in the retina include microglia, astrocytes, and Müller cells. The MGC constitutes 90% of retinal glia (Vecino et al., 2016) and has a bipolar morphology, with its nucleus located in the inner nuclear layer (INL). They contact other glial cells and different neuronal cells, allowing them to influence and be influenced by neuronal activity (García and Vecino, 2003). MGCs are located near the functional components of the retina, including the vasculature structures and the vitreous (Reichenbach and Bringmann, 2013). The stratified organization of the retina requires an extensive vascular network to meet the tissue’s metabolic demands. The outer region of the retina receives its blood supply from the fenestrated vessels of the choroid, with a nutrient exchange between the choroid and the neural retina regulated by the retinal pigment epithelium, which constitutes the outer blood-retinal barrier. Within the retina, the vasculature consists of capillary endothelial cells, pericytes, and Müller glial cells, forming the inner blood-retinal barrier (Selvam et al., 2018).

The MGC performs many retinal functions, such as energy metabolism, recycling of visual pigments, synaptic activity or neurotransmission, structural support, antioxidant effects, neuroprotection, cytokines secretion, and growth factor secretion (Reichenbach and Bringmann, 2013). Because of these functions, the MGC is considered the most critical control point for retinal homeostasis under physiological conditions.

## 2 Angiogenesis in the retina

The retina is a metabolically demanding tissue; the cone and rod photoreceptors demonstrate elevated energy metabolism, characterized by increased oxygen and glucose consumption. This heightened metabolic activity is primarily driven by ion transport processes and synaptic transmission, particularly in dark conditions (Kooragayala et al., 2015). The retina is supplied by two independent vascular beds that ensure adequate nutrient and oxygen availability: the internal retinal vasculature (which supplies the inner retina) and the external choroidal circulation (supplying the outer retina). These vascular beds develop in parallel during the embryonic stages and are closely associated with the neuronal development of the tissue. For example, the neuronal development of the mouse retina begins on embryonic day 10 (E10) and continues until postnatal day 11 (P11). Glial cells such as astrocytes do not originate in the retina but migrate radially through the optic nerve towards the retinal periphery during E17.5 and E18.5 (Chan-Ling et al., 2009). The specificity of different neuronal subtypes also takes place during these periods, and the excitatory glutamatergic signaling pathway is structured. Even in postnatal periods, neuronal maturation continues by light-activated processes (Biswas et al., 2020). The retina’s glial and neuronal specificity and maturation overlap considerably with vascular development in this tissue (Biswas et al., 2020).

Vasculogenesis is *de novo* transient blood vessels that form at the anterior and posterior poles of the eye during fetal development. This fetal ocular vasculature consists of a hyaloid system, which passes through the humor vitreous to form the posterior tunica vasculosa lentis. Consequently, vasculature covers the posterior surface of the lens. Branches of the hyaloid artery form the vasa hyaloid propia (VHP), a system of umbrella-shaped capillaries located immediately anterior to the NR (Ash et al., 2005). In the mouse retina, the hyaloid vasculature continues to grow during the five days after birth but rapidly regresses by the end of the first postnatal week and completely disappears after the second postnatal week. In humans and rodents, the regression of the hyaloid vascular system occurs due to the apoptosis of endothelial and pericyte cells and allows the initiation of retinal vascularization through angiogenesis (Biswas et al., 2020; Tisch and Ruiz de Almodóvar, 2021). In postnatal days (P0-P8), the superficial vasculature extends radially from the optic nerve head (ONH) towards the peripheral retina over the ganglion cell (GC) layer (P0-P8), following a template established by astrocytes (Biswas et al., 2020; Stone et al., 1995). Astrocytes invade the retina prenatally, and endothelial cells follow them at P1 (Tao and Zhang, 2014). It is thought this process is triggered by the release of vascular endothelial growth factor (VEGF) from hypoxic astrocytes during development (Stone et al., 1995), which leads to the vascular growth front, driving radial migration of endothelial cells (Gerhardt et al., 2003; Stone et al., 1995).

In mouse and primate retinas, vascular networks consist of three interconnected parallel vascular plexuses that supply the metabolic needs of the inner retina: the superficial vascular plexus (SVP) lies within the ganglion cell layer and the nerve fiber layer (NFL), the intermediate vascular plexus (IVP) in the inner plexiform layer (IPL), and the deep vascular plexus (DVP) in the outer plexiform layer (OPL) (Fruttiger, 2007). At birth, the first to form is the SVP, from which bubbling vessels descend and advance to depth to establish the DVP in the OPL at P8-P12, guided by MGC processes (Biswas et al., 2020; Stone et al., 1995). Eventually, the vessels ascend and advance within the IPL layer to form the IVP (starting at P12) and complete vascularization by P21 (Son et al., 2020; Stahl et al., 2010). Interneurons have also been reported to be involved in developing DVP and IVP (Usui et al., 2015).

Thus, the specification of neuronal and glial fates and the establishment of neuronal activity in the retina overlap with the development of vessels in this tissue, with glial involvement regulating their proper establishment (Biswas et al., 2020).

### 2.1 The glial contribution to retinal angiogenesis in physiology: a role for Müller glial cells

Glial cells are essential regulators of retinal angiogenesis, secreting pro- and anti-angiogenic factors necessary for this process. In the mammalian retina, vascular endothelial cells are always accompanied by astrocytes. Areas of the retina with low vascular density have few astrocytes, whereas avascular areas lack these glial cells (Schnitzer, 1988a, 1987). In humans and guinea pigs, the foveal zone is completely avascular and devoid of astrocytes (Ikui et al., 1976; Small et al., 1991), whereas, in rabbits, astrocytes are restricted to a small, unique vascularized zone called the medullary rays (Schnitzer, 1988a, 1988b).

An essential factor that regulates retinal angiogenesis is the VEGF (Gerhardt et al., 2003; Stone et al., 1995). The concentration of this protein increases in the retina during embryonic development and gradually decreases in the postnatal stages (Famiglietti et al., 2003). Knockout mouse models have shown that deficiency of this growth factor leads to abnormal development of blood vessels and a lethal phenotype (Carmeliet et al., 1996; Ferrara et al., 1996). VEGF is produced by several cell types of the mouse NR, mainly astrocytes and MGC. Using cell-specific deletions of the *Vegfa* gene by the Cre/LoxP system, it was shown that the absence of this growth factor in astrocytes altered the growth of the SVP. In contrast, deleting *Vegfa* in neurons and MGC impaired DVP growth without altering SVP growth (Biswas et al., 2020; Rattner et al., 2019). These data indicate that VEGFA derived from glial sources contributes to developing and maintaining various vascular plexuses in the retina (Biswas et al., 2020; Stone et al., 1995).

In the retina, the Norrin-Frizzled-4 (Fzd4)-low-density lipoprotein receptor-related protein 5 (Lrp5)-tetraspanin 12 (Tspan12) signaling module is closely associated with angiogenesis. MGC mainly secretes Norrin in the retina and binds to its high-affinity receptor Fzd4, which is expressed in neurons and endothelial cells (Ye et al., 2009). Disruption of the Norrin pathway in the retina, either genetically or by neutralizing antibodies, results in loss of retinal vascular integrity (Paes et al., 2011; Wang et al., 2012), delay of SVP growth, and loss of DVP (Chen et al., 2012; Luhmann et al., 2005; Ye et al., 2009). These studies suggest that MGC-derived factors, such as VEGF and Norrin, are crucial for retinal angiogenesis and play a predominant role in DVP formation and a partial role in SVP during angiogenic vascular development stages.

## 3 Formation of the blood-retinal barrier

**Figure 2:**
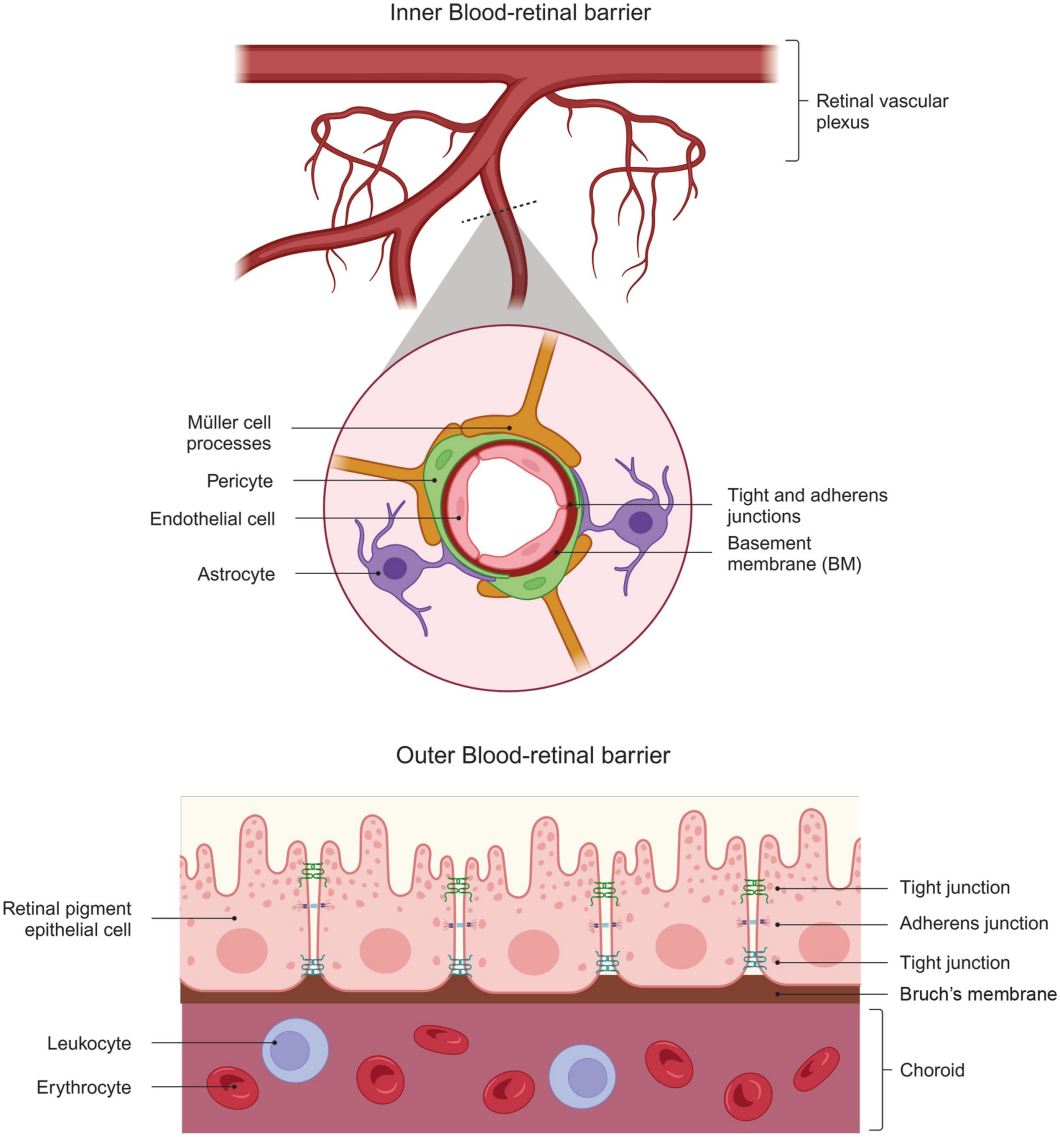
Structural Complexity of the Blood-Retinal Barrier. The diagram shows the structure of the inner blood-retinal barrier (top), which consists of endothelial cells, pericytes, astrocytes, and the processes of MGC. The outer blood-retinal barrier (bottom) comprises the retinal pigment epithelium cells that limit the choroid’s passage. Both barriers comprise elements that give them part of their barrier properties: tight and adherent junctions.

The blood-retinal barrier (BRB) is a “gliovascular unit” in which macroglial cells surround capillary endothelial cells and regulate them through paracrine interactions (Abukawa et al., 2009). The mammalian retina has two types of BRBs: the outer BRB (oBRB) and the inner BRB (iBRB). The oBRB primarily comprises tight junctions between the retinal pigment epithelial cells and its underlying basement membrane (BM) (Burns et al., 2021). The oBRB separates the photoreceptors from the choroidal circulation (Runkle and Antonetti, 2011) and consists of a highly fenestrated vascular bed (Saint-Geniez et al., 2008) (Figure 2). Expression of VEGF is vital for its maintenance, and its absence in the RPE leads to failure of choroidal development and loss of visual function (Marneros et al., 2005).

On the other hand, the iBRB is a complex unit composed of the tight junctions of retinal vascular endothelial cells and supported by pericytes, astrocytes, MGC, microglia, neurons, and extracellular matrix proteins (Cunha-Vaz et al., 2011). This composition allows the iBRB to exhibit characteristics of both paracellular and transcellular barriers and acts as an interface between the surrounding blood and the neural retina with selective permeability (Abukawa et al., 2009). The characteristics of the paracellular barrier are established when endothelial cells form tight junctions that prevent the movement of small molecules between them.

In rodents, the iBRB is established between P3 and P5 days in the central retina SVP. By P10, the maturation of the iBRB as a “gliovascular unit” with paracellular and transcellular barrier properties in the deep and superficial vascular beds is complete. In contrast, the intermediate plexus (the last of the three plexuses to be formed) has had an intact BRB since the beginning (Chow and Gu, 2017). Both the transcellular and paracellular barriers reach maturity between P10 and P18 days (Chow and Gu, 2017).

### 3.1 Müller cells in the inner blood-retinal barrier formation

As mentioned earlier, glial cells are essential regulators in retinal angiogenesis, and MGC, in particular, are involved in the structural organization of the BRB (García and Vecino, 2003). Blood capillaries are lined by the processes of MGC, which serve as a communicating system for metabolic exchange between the vasculature and neurons (García and Vecino, 2003). One of the characteristic proteins in these barriers is Aquaporin-4 (AQP4). In the retina, this protein is expressed in the end-feet of astrocytes surrounding the SVP and in MGCs in contact with the DVP (Nicchia et al., 2016). Studies using AQP4 knockout mice show vascular leakage only in the DVP, suggesting that expression of this protein in MGC but not astrocytes is critical for maintaining BRB integrity (Nicchia et al., 2016).

An interesting study showed that implanting MGC in the anterior chamber of rats’ eyes resulted in the formation of new blood vessels, indicating a close relationship between vessel formation and MGC’s ability to regulate their functions (Tout et al., 1993). On the other hand, the relationship between endothelial cells and MGC has been the subject of studies in recent years, which will be discussed in the following sections.

### 3.2 Müller cell interactions with endothelial cells

The interactions between MGC and other cell members of iBRB, such as endothelial cells, have been studied mainly using *in vitro* models, in which these cells are co-cultured to understand their involvement and linkage in physiology and pathology. In these studies, MGC has been hypothesized to maintain an “anti-angiogenic background” under physiological conditions (Yafai et al., 2007), significantly limiting events such as angiogenesis.

The anti-angiogenic effect is evident when endothelial cells are co-cultured with MGC *in vitro*. MIO-M1 cells (Moorfields/Institute of Ophthalmology-Müller 1), a human immortalized MGC line, induce an anti-angiogenic state in bovine retinal endothelial cells (BRECs), resulting in an increase in proliferation and a decrease in apoptosis, and cell migration rates (Yafai et al., 2004), and cell permeability (Tretiach et al., 2005, 2004). Moreover, when BREC cells were exposed to a conditioned medium of MIO-M1 cells, cell proliferation and ERK1/2 activity decreased, independent of their activation by VEGF (Yafai et al., 2007, 2004). These studies demonstrate the ability of MGC, which, together with endothelial cells, to maintain control over retinal angiogenesis, partly mediated by the release of angiogenic and anti-angiogenic factors.

## 4 The role of Müller glial cells: angiogenic imbalance and vasoproliferative retinopathies

Angiogenesis is critical for the development and function of many tissues, including the retina (Folkman, 2006). The relative balance between pro- and anti-angiogenic molecules accurately controls angiogenic homeostasis (Folkman, 1995). However, this balance is disturbed under neovascular pathological conditions (Eichler et al., 2004a). When this occurs, angiogenesis is increased and contributes to the development of vitreo-retinal vascular diseases (Cappelli et al., 2021). Some of the retinal pathologies in which angiogenesis is a critical event include diabetic retinopathy, retinal vein occlusion, retinopathy of prematurity, and age-related macular degeneration (Cai et al., 2012). Angiogenesis includes different stages involving proliferation, chemotaxis, migration of microvascular endothelial cells, and metabolism of the extracellular matrix (Eichler et al., 2004a). This series of events is regulated by growth factors, cytokines, their associated cellular receptors, extracellular matrix components, and environmental factors such as hypoxia (Eichler et al., 2004b). It is known that the release of pro-angiogenic factors from MGC can increase vascular damage in the retina (Bringmann et al., 2006).

Currently, VEGF and VEGF receptor 2 (VEGFR2) are considered to be the primary mediators of angiogenesis (Carmeliet and Jain, 2011). However, other molecules are also secreted by MGC with pro- or anti-angiogenic activity. Pro-angiogenic molecules of MGC include essential fibroblast growth factor (bFGF), insulin-like growth factor (IGF), angiopoietin-like 4 (ANGPTL4), endothelial growth factor (EGF), and placental growth factor (PlGF) (Eichler et al., 2004a, 2004b). Meanwhile, known anti-angiogenic factors include pigment-epithelium-derived factor (PEDF), TGF-*β*2, and thrombospondin-1 (TSP-1) (Eichler et al., 2004a, 2004b).

### 4.1 Retinal Müller cell angiogenic secretome

In the neural retina, MGC has been described as the cellular entity responsible for releasing angiogenic mediators that play an essential role in neuro-retinal vascular homeostasis. Accordingly, VEGF plays a central role in vascular development and homeostasis. This factor is a polypeptide secreted by various tissues in response to hypoxia and activates its receptors on endothelial cells to promote survival, proliferation, permeability, and cell migration (Chung and Ferrara, 2011). Molecular and environmental mediators positively regulate its expression and release, including hypoxia, inflammation, excitotoxicity, oxidative stress, and hyperglycemia (Behl and Kotwani, 2015). Under these conditions, *Vegf* gene transcription is controlled by hypoxia-inducible factor (HIF) proteins and *α*-β heterodimeric transcription factors that are stabilized by tissue hypoxia and act as intracellular oxygen sensors (Semenza, 2014).

In the neural retina, MGCs are the predominant source of VEGF (Fu et al., 2015; Saint-Geniez et al., 2008; Wang et al., 2015). The angiogenic effects of VEGF are mediated by VEGFR2, which is expressed in all retinal cells but is more prominent in MGC and retinal endothelial cells (Saint-Geniez et al., 2008; Simmons et al., 2018). Both cells increase VEGFR2 expression in response to VEGF (Rossino et al., 2020). However, the type and duration of VEGF stimulus predict the neuroprotective or neurodegenerative behavior of GMCs (Bai et al., 2009; Jiang et al., 2014; Subirada et al., 2018). In addition, MGC-derived VEGF may exert an autocrine loop that promotes the expression of more retinal VEGF (Rossino et al., 2020). The angiogenic effects of VEGF are mediated by VEGFR2, which is expressed in all retinal cells but is more prominent in MGC and retinal endothelial cells (Kim et al., 1999; Saint-Geniez et al., 2008; Simmons et al., 2018). Both cells increase VEGFR2 expression in response to VEGF (Rossino et al., 2020). Temporal expression of endogenous VEGF promotes axonal growth and post-ischemia and aids in reorganizing glial processes within retinal tissue (Hayakawa et al., 2011; Hermann and Zechariah, 2009; Storkebaum and Carmeliet, 2004). However, the type and duration of VEGF stimulus predict the neuroprotective or neurodegenerative behavior of GMCs (Bai et al., 2009; Jiang et al., 2014; Subirada et al., 2018).

Moreover, hypoxia upregulates numerous factors, including EGF, which may contribute to the induction of abnormal angiogenesis and damage the structural arrangement of BRB. Stimulation of EGF receptors (EGF-R) in MGC activates proliferative and migratory behavior, often leading to progressive and permanent vision loss. *In vitro* experiments have demonstrated a link between VEGF and EGF in MGC. Exposure to exogenous VEGF increases the expression of EGF-R in rat MGC lines (rMC-1) to an even greater extent than the expression induced by its cognate ligand, EGF (a 20-fold increase compared with a 3-fold increase) (Peña and Vazquez, 2020). In the retina, paracrine signaling of VEGF has been linked to the upregulation of heparin-binding EGF ligand (HB-EGF) after injury, activating through EGF-R (Peña and Vazquez, 2020). Upregulation of EGF-R via HB-EGF stimulates MGC to initiate dedifferentiation responses via wnt/*β*-catenin (Wan et al., 2012).

In vasoproliferative retinopathies, rupture of the BRB and occlusion of the micro-capillaries are common events. In response to this event, MGC increases the secretion of pro-angiogenic factors, such as VEGF and fibroblast growth factor (FGF) (Peña and Vazquez, 2020). bFGF can induce proliferation, migration, and extracellular matrix proteolysis in endothelial cells (Yafai et al., 2013), whereas FGF2 activates proliferation and gliotic responses in MGC (Hollborn et al., 2004), and induces expression of proinflammatory genes in MIO-M1, and seems to play a significant role in activation of MGC during DR (Rezzola et al., 2021). In this disease, FGF2 has been detected in epiretinal membranes formed by gliotic events of MGC (Hueber et al., 1997).

In addition to VEGF, HIF upregulates factors such as ANGPTL4 in MGC *in vitro* under hypoxic conditions and *in vivo* in the ischemic inner retina (Xin et al., 2013). Secretion of ANGPTL4 has been shown to modulate the disposition of circulating triglycerides by inhibiting lipoprotein lipase (Yin et al., 2009). ANGPTL4 may promote disruption of vascular integrity by sequentially interacting with integrin α5*β*1, vascular endothelial-cadherin, and claudin-5 (Huang et al., 2011).

The anti-angiogenic factors antagonize the angiogenic process in different tissues; the PEDF is the primary inhibitor of angiogenesis in the retina (Dawson et al., 1999). PEDF is a glycoprotein first identified in the culture medium of RPE cells (Tombran-Tink and Johnson, 1989) and is now known to block the migration and proliferation of endothelial cells (Duh et al., 2002). High levels of PEDF have been detected in ocular compartments such as the cornea and vitreous, indicating its critical role in maintaining local avascularity (Karakousis et al., 2001). In the neural retina, MGCs are essential producers of PEDF (Eichler et al., 2004a). The anti-angiogenic mechanism of PEDF involves blocking the angiogenic signaling pathway activated by VEGF, specifically nuclear translocation of HIF-1, and inhibiting the phosphorylation of ERK1/2, a pathway involved in retinal angiogenesis. This mechanism has been described in retinal capillary endothelial (RCEC) cells (Zhang et al., 2006).

In contrast, in microvascular diseases like diabetic retinopathy, PEDF levels in the vitreous and aqueous humor decrease markedly. This seems to be closely related to the progression of the retinopathy (Boehm et al., 2003; Ogata et al., 2002, 2001). Exogenous administration of PEDF significantly inhibited neovascularization in animal models of ischemic retinopathies (Duh et al., 2002; Stellmach et al., 2001), possibly by inducing apoptosis in the endothelial cells of the new vessels (Chen et al., 2006; Volpert et al., 2002). Experimental hypoxia models induce overexpression of VEGF and reduction of PEDF in MGC (Eichler et al., 2004a; Yafai et al., 2007). In addition, PEDF has neurotrophic and neuroprotective functions in the retina (Cao et al., 2001, 1999; Ogata et al., 2001; Takita et al., 2003). The anti-angiogenic mechanism of PEDF involves blocking the angiogenic signaling pathway activated by VEGF, specifically nuclear translocation of HIF-1, and inhibiting the phosphorylation of ERK1/2, a pathway involved in retinal angiogenesis. This mechanism has been described in BREC cells (Bullard et al., 2003; Hutchings et al., 2002; Zhang et al., 2006). In NR, the MGC is the primary source of both VEGF and PEDF (Fu et al., 2015; Saint-Geniez et al., 2008; Unterlauft et al., 2014; Wang et al., 2010). This balance between pro- and anti-angiogenic factors, which exist under healthy physiological conditions, is disrupted during pathological events.

The mammalian TGF-β family includes TGF-β1, TGF-β2, and TGF-β3. TGF-β2 is a dimeric polypeptide that regulates cellular growth, proliferation, apoptosis, differentiation, extracellular matrix synthesis, and immunosuppression (Fuchshofer et al., 2005; Lu et al., 2004; Pardali et al., 2010; Roberts and Sporn, 1993). MGC produces substantial amounts of TGF-β2, inhibiting the proliferation of retinal microvascular endothelial cells (Eichler et al., 2004b, 2001). This factor binds to the serine/threonine kinase type II TGF-β2 receptor located in BRECs, which activates and phosphorylates Smad2 and Smad3, attenuating the phosphorylation state of ERK1/2. Thus, TGF-β2 released by MGC has anti-angiogenic activity by inhibiting the proliferation of retinal endothelial cells (Yafai et al., 2014). It has also been described that TGF-β2 can exert anti- or pro-angiogenic effects depending on the cellular conditions, with the anti-angiogenic being maintained under a physiological environment (Yafai et al., 2014).

TSP-1, a sizable multi-domain glycoprotein of the extracellular matrix, can inhibit angiogenesis by inducing apoptosis in endothelial cells (Jiménez et al., 2000); therefore, TSP-1 is involved in the anti-angiogenic background effect in the healthy retina. Furthermore, TSP-1 may render endothelial cells refractory to important angiogenic inducers such as VEGF. The mechanism of TSP-1 depends on its binding to the CD36 receptor, which results from the interaction of upregulated FasL with CD95/Fas and its subsequent apoptosis induction in endothelial cells (Jiménez et al., 2000). TSP-1 also competes with FGF for binding to the endothelial cell surface (Eichler et al., 2004b).

**Figure 3:**
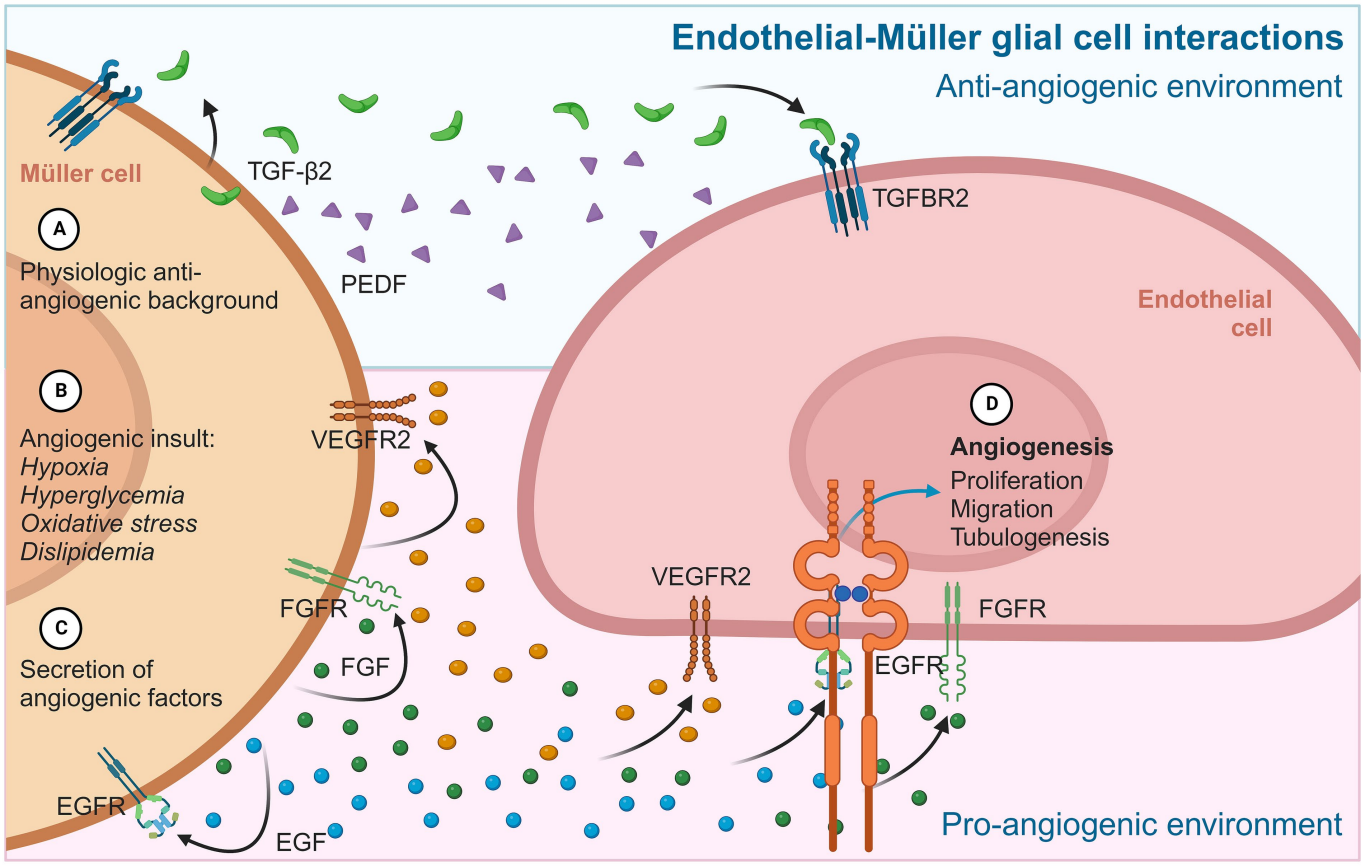
Diagram summarizing the main angiogenic interactions between MGC and endothelial cells. In physiological contexts **(A)**, MGC provides a permanent anti-angiogenic environment to retinal endothelial cells, mainly through PEDF and TGF-β2. In an angiogenic stimulus **(B)**, MGC adopts an active gliotic phenotype that prioritizes cytokine secretion **(C)** and pro-angiogenic factors such as VEGF, FGF, and EGF. These proteins are released from MGC and have autocrine/paracrine effects on neighboring MGC, which maintain the gliotic and reactive phenotype, and on iBRB endothelial cells, inducing typical angiogenic events **(D)** such as proliferation, migration, and the formation of tubular structures.

Although the identities and functions of many factors and cytokines that modulate angiogenesis are still unknown, glial cells such as MGC are evidently involved in regulating vascular homeostasis and related processes such as inflammation by releasing protein factors that regulate the activity and behavior of various cells, such as retinal endothelial cells (Figure 3).

### 4.2 Diabetic retinopathy

Diabetic retinopathy (DR) is the leading cause of adult blindness in the working-age population in industrialized countries (Biswas et al., 2020). DR is characterized in its early stages by microvascular damage, capillary occlusion, and angiogenesis in the proliferative diabetic retinopathy stage (Yafai et al., 2004). An increase in VEGF has been reported as an essential marker of disease progression and the occurrence of events related to angiogenesis and BRB disruption (Aiello et al., 1994; Malecaze et al., 1994). DR is considered a multifactorial disease, with processes such as hyperglycemia, oxidative stress, inflammation, hypoxia, and dyslipidemia contributing to its development (Al-Kharashi, 2018).

Transcriptomic studies performed on the retinas of patients with DR and healthy individuals have identified upregulated genes, particularly those related to inflammatory responses and angiogenesis (Tang and Kern, 2011). The highly neovascular phenotype in DR reflects an imbalance between angiogenic and anti-angiogenic factors. In the vitreous of patients with DR, there is a decrease in PEDF and an increase in VEGF (Ogata et al., 2002, 2001). This increase in VEGF is observed *in vitro* in MGC cultures exposed to hyperglycemic or hypoxic conditions (Fletcher et al., 2007). Moreover, treatment with intravitreal injections of PEDF for 4 weeks in streptozotocin-induced diabetic rats ameliorates the diabetes-induced changes such as a decrease in a- and b-wave amplitude in electroretinogram, overexpression of glial fibrillary acidic protein (GFAP) in MGC, increase in VEGF, and disruption of BRB (Yoshida et al., 2009).

On the other hand, the pathological changes in the retina during PDR, activated MGC may progress to reactive gliosis, a nonspecific reactive response of glial cells to damage characterized by uncontrolled proliferation, migration, and increased expression of gliosis markers (Bringmann et al., 2006). In addition, MGC can undergo fibrotic transdifferentiation, which contributes to the formation of fibrovascular epiretinal membranes (ERM) that can exert tractional forces on the retinal surface and cause retinal detachment (Oberstein et al., 2011; Roy et al., 2016). In diabetic mouse retinas, ERM formation was stimulated by TGF-*β*1 through the PI3K/Akt signaling pathway (Zhang and Kong, 2020). These glial-derived structures have low PEDF levels (Lange et al., 2008), which are inversely correlated with the degree of retinal neovascularization (Gao et al., 2001), and present positivity for molecular markers related to glial-mesenchymal transition, including SNAIL proteins, smooth muscle proteins, VEGFA and TGF-β receptors (Wu et al., 2020).

Placental Growth Factor (PlGF), a member of the VEGF family, is also detected at high levels in vitreous PDR patients (Mitamura et al., 2002). PlGF is significantly released from the MGC and plays a physiological role in cellular survival. Its dysregulation may correlate with the development of DR (Sensenbrenner, 1993). In contrast, a deficiency of PIGF in diabetic mice prevents BRB breakdown and retinal cell death (Huang et al., 2015).

Most studies have focused on animal models of type 1 DM, although the highest prevalence of diabetes worldwide is type 2. The Goto-Kakizaki (GK) rat is a genetic model of non-obese type 2 diabetes that develops glucose intolerance corresponding to insulin resistance (Hachana et al., 2020). In this animal model, adult rats have abundant lipid deposits in Bruch’s membrane (Kimura et al., 1982). This is consistent with humans with type 2 DM exhibiting increased serum concentrations of various fatty acids associated with disease progression (Korani et al., 2012). RD is activated by cellular damage in the retina stimulated by the diabetic environment, including increased levels of intraocular free fatty acids. Free fatty acids may serve as triggers for releasing inflammatory cytokines from MGC. The resulting cytokines may be potent stimulators of retinal endothelial pathologies, such as leukostasis, vascular permeability, and basement membrane thinning (Capozzi et al., 2020). In humans, MGC exposed to fatty acids such as linoleic acid and oleic acid, production of VEGF, interleukin-6 (IL-6), and IL-8 was observed. This response was not recapitulated in human astrocytes or retinal pigment epithelial cell lines (ARPE-19). Other fatty acids, such as palmitic acid, have also shown a robust angiogenic response in the mouse retina, blocked by inhibition of the PPARβ/*δ* receptor (Capozzi et al., 2020). These reports suggest specific mechanisms of MGC for producing pro-angiogenic factors in response to lipid molecules, which are vital for the progression of DR (Capozzi et al., 2016).

In current treatments of DR, VEGFA activity is neutralized by intravitreal administration of anti-VEGF antibodies such as ranibizumab or aflibercept (Cai et al., 2021). A significant increase in apoptosis and autophagy has been observed in MGC treated with these drugs (Le, 2017; Saint-Geniez et al., 2008). This complements recent evidence that the VEGF released by MGC protects itself and retinal neurons (Le, 2017; Matsuda et al., 2017) and acts as a neuroprotective factor when adverse conditions threaten neuronal survival (Amato et al., 2016). Moreover, disruption of VEGFR2 in rat MGC and mouse retina under diabetic conditions leads to apoptosis of these cell types (Fu et al., 2015; Saint-Geniez et al., 2008). This effect might be different in the MIO-M1 cell line (Matsuda et al., 2017).

In recent years, vitreous humor from patients with PDR in retinal cell cultures has been used as an *in vitro* model of DR. In MIO-M1 cells, applying vitreous obtained from PDR patients increased cell proliferation, migration, and expression of proinflammatory cytokines and chemokines (Rezzola et al., 2021). Surprisingly, this inflammatory phenotype did not appear to be mediated by stimulation with VEGF or bFGF. Anti-VEGF treatments such as ranibizumab had no protective effect. However, using FGFR receptor antagonists (BGJ398) and anti-FGF traps (NSC12) inhibited the proinflammatory responses induced by the vitreous PDR patients (Rezzola et al., 2021). Exosomes expressing the microRNA miR-9-3p, derived from MGC, and whose described mechanism involves binding to coding sequences of the sphingosine-1-phosphate receptor S1P1 in endothelial cells. miR-9-3p were identified in the vitreous of PDR patients, which subsequently activates VEGFR2 phosphorylation and internalization in the presence or absence of exogenous VEGFA, inducing proliferation, migration, and tube-forming ability in primary human retinal endothelial cells (hREC) (Liu et al., 2022). Other studies have found increased levels of PCED1B Antisense RNA 1 (PCED1B-AS1) in the vitreous of DR patients (Wang et al., 2022). In *in vitro* cultures of MGC exposed to hypoxic or hyperglycemic conditions induced by CoCl_2_ or high glucose, respectively, PCED1B-AS1 significantly attenuated the upregulation of VEGF and MCP-1. It also attenuated VEGF-induced proliferation and migration of HRMECs (Wang et al., 2022). Information suggests that both are markedly released in MGC and play a promising role in treating DR (Liu et al., 2021; Patel et al., 1994).

Hypoxic conditions promote a significant increase in pro-angiogenic factors in MGC, including VEGF and ANGPTL4, leading to angiogenesis and increased vascular permeability of endothelial vascular cells (Ou et al., 2019). Both *in vitro* and *in vivo*, the upregulation of both proteins in MGC is regulated by HIF-1 (Babapoor-Farrokhran et al., 2015). ANGPTL4 expression was also increased in the vitreous and aqueous humor of PDR patients and localized mainly in areas of retinal angiogenesis. ANGPTL4 has been associated with promoting vascular permeability and macular edema in diabetic patients. Inhibition of ANGPTL4 expression reduced the angiogenic potential of hypoxic MGC, and this effect was additive with inhibition of VEGF expression (Babapoor-Farrokhran et al., 2015). Another factor associated with these hypoxic conditions is bFGF. Ischemia–reperfusion or post-ischemia conditions in rat retinas increased bFGF levels in MGC or isolated MGC *in vitro*. In PDR patients, bFGF immunoreactivity colocalizes with GFAP-positive cells in surgically removed retinal tissues. The cells synthesize mRNA and the bFGF protein and release it, which is increased under hypoxia or oxidative stress and stimulated by growth factors and cytokines, including pro-inflammatory factors. The conditioned culture medium of cells exposed to hypoxia for 72 h had a stimulatory effect on retinal endothelial cell proliferation and ERK1/2 phosphorylation. Treatment with neutralizing bFGF antibodies suppressed both effects similarly to anti-VEGF antibodies (Yafai et al., 2013).

Many of these pathologic effects appear to be a side effect of the increased expression of pro-angiogenic and pro-inflammatory cytokines and growth factors in the diabetic retina, mainly promoted by the active response of MGC.

### 4.3 Retinopathy of prematurity

The retina is among the last organs to undergo vascularization during fetal development, initiating around 16 weeks’ gestation. At this stage, vasculogenesis starts in the posterior region of the superficial retina, precisely the optic disk, and extends towards the periphery. By 25 weeks gestation, angiogenesis begins from the optic disk and progresses peripherally, developing a deeper vascular network. This process is driven by the hypoxic uterine environment, which induces pro-angiogenic growth factors that promote retinal blood vessel growth to meet the increasing oxygen demands of the developing retina. Complete retinal vascularization is achieved by approximately 36–40 weeks’ gestation. The relatively hyperoxic postnatal environment then prevents further vasoproliferation (Koc and Bas, 2024; Tsang et al., 2019).

Retinopathy of prematurity (ROP) is the most common cause of visual impairment in infancy worldwide and causes 170,000 infants to go blind annually around the world (Hartnett and Penn, 2012; Hoppe et al., 2020). It occurs primarily in premature infants because of high oxygen concentrations in incubators (Park et al., 2010). ROP is a two-stage ocular disease, and the first stage is vaso-obliteration caused by hyperoxia due to excessive oxygen consumption to support respiratory function (Mutlu and Sarici, 2007). Delayed growth of retinal vessels and degeneration of preexisting vessels are hallmarks of this stage. The second stage is vasoproliferative, triggered by relative hypoxia caused by the retinal vessels not carrying enough oxygen (Smith, 2003). The second stage is abnormal retina neovascularization at the interface between avascular and vascularized areas (Cai et al., 2021). The severity of retinopathy of prematurity (ROP) is inversely related to gestational age. Since retinal vascularization is typically completed around birth, premature infants often exhibit a larger area of retinal avascularity. Postnatally, exposure to a hyperoxic environment exacerbates vasoattenuation of the sparse retinal vasculature. When these infants are subsequently returned to a normoxic room air environment, retinal hypoxia ensues, leading to the development of ROP (Penn, 2008).

MGC produces TGF-*β*2 under physiological and post-ischemic conditions. In MGC cultures with BRECs, TGF-β2 is secreted by MGC bound to TGF-β receptors in BRECs and activated anti-proliferative signaling pathways (Yafai et al., 2014). In the immortalized human MGC line, MIO-M1, and guinea pig MGC, hypoxia resulted in decreased release of TGF-β2 and PEDF but increased release of TSP-1. All three factors inhibited the proliferation of retinal endothelial cells. It is also possible that hypoxia-induced VEGF stimulates the production of TSP-1 in MGC, inhibiting endothelial cell growth via a negative feedback mechanism (Eichler et al., 2004b).

In the retinas of a rat ROP model, the oxygen-induced retinopathy (OIR) model, VEGFA mRNA was found mainly in the INL corresponding to CRALBP-positive MGC (Wang et al., 2013). In this model, specific knockdown of MGC-derived VEGF164 by short hairpin RNAs maintained long-term inhibition of intravitreal neovascularization and limited cell death (Jiang et al., 2014) without affecting retinal vascular development and reducing VEGFR2 phosphorylation in retinal endothelial cells (Wang et al., 2013). An *in vitro* iBRB model co-culture mouse microvascular endothelial cells and MGC exposed to hypoxia showed that the response to the oxygen deprivation is more harmful in endothelial cells, compared with the iBRB model without MGC, resulting in more impaired barrier function in the iBRB *in vitro* model (Inada et al., 2021). This suggests that communication between MGC and endothelial cells requires highly orchestrated mechanisms in which MGC can act as sensors of retinal damage.

### 4.4 Age-related macular degeneration

Angiogenesis is a complex process for vascular development and pathologic conditions such as age-related macular degeneration (AMD). Intraretinal and subretinal neovascularization originates from the inner retinal vessels in a subtype of AMD called retinal angiomatous proliferation (RAP) (Dorrell et al., 2009). RAP is one of the most common causes of blindness worldwide (Miller, 2013). It arises from superficial vascular plexus (SVP) capillaries and develops initially intraretinal (stage I), subretinal (stage II), and finally to choroidal neovascularization and retinal-choroidal anastomosis (stage III) (Wei et al., 2019). Pathologically, RAP is an intraretinal angiomatous complex in the outer retina (Monson et al., 2008; Wei et al., 2019). Few animal models simulate RAP. In a mouse model of RAP, which has characteristics like human patients and involves deletion of the very low-density lipoprotein receptor (VLDLR), MGC was activated near the lesion and showed increased expression of VEGF and bFGF. In the retinas of this model, there was also an increase in phosphorylation of Akt and MAPK and translocation of NFkB (Li et al., 2007). Recently, a model involving intravitreal administration of DL-2-aminoadipic acid (DL-AAA), an inhibitor of glutamine synthetase (GS) toxic to MGC, was described. After 8 to 12 weeks, administration of DL-AAA in rabbits resulted in intraretinal detachment with retinal degeneration, retinal and choroidal inflammation, retinal vascular leakage, retinal hemorrhage, dilated retinal vessels, and retinal angiogenesis with epiretinal vessels growing toward the peripheral retina and central area. The mechanism by which DL-AAA produces these effects has yet to be fully understood. However, it has been hypothesized that DL-AAA alters the connections between BRB, causing retinal metabolic dysfunction, retinal ischemia, and inflammation, resulting in high concentrations of VEGF that induce a chronic state of neovascularization (Kumar et al., 2022). Interestingly, in this animal model, the phenotype RAP is achieved by direct changes in MGC and their physiological function by promoting its secretory phenotype of proangiogenic factors that stimulate retinal angiogenesis (Kumar et al., 2022; Mao and Liu, 2013; Sapieha et al., 2010).

## 5 Müller glial cells as a therapeutic target for vasoproliferative retinopathies: future research

Current treatments for vasoproliferative retinal diseases, such as diabetic retinopathy, are based on panretinal photocoagulation and anti-VEGF therapy (Régnier et al., 2014). Panretinal photocoagulation involves laser-induced peripheral retinal scarring of neovascular tissue, a nonspecific treatment that may damage viable non-vascular retinal cells, temporarily halting the progression of neovascularization. In contrast, anti-VEGF therapy relies on applying monoclonal antibodies targeting VEGF-A to neutralize downstream signaling, thereby preventing the progression of angiogenesis in diabetic retinas (Behl and Kotwani, 2015). Despite being widely recognized as the gold standard for treating PDR, different studies have shown that anti-VEGF therapy is ineffective in a significant proportion of patients, and its efficacy diminishes over time in another considerable percentage of PDR patients (Bressler et al., 2013; Ip et al., 2012). Given that VEGF-A is a neuroprotective cytokine in retinal neurons, several studies have indicated that indiscriminate inhibition with anti-VEGF-A antibodies can lead to a loss of retinal neurons (Amato et al., 2016; Saint-Geniez et al., 2008), suggesting that other more specific treatments should accompany anti-VEGF therapy.

The discovery that MGC is the primary source of VEGF-A release in the neuroretina has sparked an interest in investigating whether therapies targeting these cells could help halt or limit the progression of vasoproliferative diseases, such as proliferative diabetic retinopathy (PDR), retinopathy of prematurity (ROP), and age-related macular degeneration (AMD). We summarize and propose key considerations that may guide biomedical research to establish MGC as a therapeutic cellular target in vasoproliferative retinopathies.

### 5.1 Exploration of other angiogenic molecules and specific regulation of cytokine and growth factor release by Müller glial cells

VEGF has been described as one of the main pro-angiogenic factors; however, several studies over the past few decades have identified several pro-angiogenic and anti-angiogenic molecules, many of which are released by MGC. These include ANGPLT4, PEDF, BDNF, and Angiopoietin-2, among others (Babapoor-Farrokhran et al., 2015; Calado et al., 2016; Gong et al., 2012; Seki et al., 2004; Striglia et al., 2020). Novel therapies for treating vasoproliferative retinopathies could involve modulating the natural release of cytokines or growth factors from MGC to shift pro-angiogenic and neurodegenerative retinal microenvironments toward anti-angiogenic, neuroprotective, and pro-survival states. At this moment, it is crucial to understand in detail the molecular mechanisms of anti-angiogenic proteins or growth factors, not only in MGC but also in other retinal cell players. Understanding the specific expression of the release system and receptors that modulate the downstream release of growth factors and cytokines will enable pharmacological or genetic interventions to block excessive release without compromising the function of other glial or non-glial cells. In this regard, the extended localization of MGC, adjacent to the vitreous and subretinal space, makes it one of the first cell types to respond to intravitreal or subretinal injections (Devoldere et al., 2019), thus facilitating the delivery of specific drugs that modulate the synthesis and release of angiogenic proteins. Moreover, numerous studies show that neural retinal damage occurs before detectable vascular damage (Sinclair and Schwartz, 2019; Verma et al., 2011), suggesting that modulating the pro- and anti-angiogenic environment of MGC could influence neuronal, glial, and retinal endothelial survival, damage, or death, not only in proliferative stages of DR but also at an early stage.

### 5.2 Extracellular vesicles (EVs)

EVs are a less-explored molecular tool in Müller cells and the retina. They are nano-sized vesicles secreted into the extracellular environment by all forms of living cells. They are essential elements in cell-to-cell communication and carry bioactive molecules such as amino acids, proteins, RNA, and lipids. Substantial evidence suggests that EVs can modulate angiogenesis in organs and have potential therapeutic applications (Todorova et al., 2017). However, compared to other organs and tissues, the available information regarding angiogenic mechanisms, biogenesis, and cargo of EVs in the retina is considerably limited.

Nevertheless, there is evidence that Müller cells secrete EVs capable of modulating angiogenesis in endothelial cells (Liu et al., 2022). There is also evidence that EVs loaded with anti-VEGF molecules can reduce the frequency of intravitreal injections for the treatment of DR in an animal model (Reddy et al., 2023). Further research is needed to determine their protein and non-protein cargo and how these molecules might modulate angiogenesis in retinal tissues. Understanding these mechanisms will enhance our ability to modulate them in various vasoproliferative pathologies through EV-based therapies.

### 5.3 Heterogeneity within the Müller glial cell population

In the last decade, heterogeneity within the MGC population has begun to be explored (Roesch et al., 2008; Syrbe et al., 2018; Voigt et al., 2019). This heterogeneity has been reported regarding MGC localization within the retina, which also influences the cellular morphology, physiology, and expression of several proteins, such as the enzyme GS and glutamate transporter (GLAST), structural cytoskeletal proteins like GFAP, calcium channels, and several transcription factors (Bringmann et al., 2018; Distler and Dreher, 1996; Matet et al., 2015; Reichenbach and Bringmann, 2013, 2020; Nishikawa and Tamai, 2001; Roesch et al., 2008; Vecino et al., 2016). Recently, MGC heterogeneity has also begun to be explored in their cellular response in animal models of OIR and DR (Deng et al., 2024; Yao et al., 2024). However, this heterogeneity has not yet been studied in terms of the MGC’s ability to secrete specific cytokines or growth factors and, in turn, MGC’s ability to induce retinal angiogenesis in the early stage of the disease. This heterogeneity also affects the points above, potentially determining specialization in the upstream and downstream mechanisms involved in the release of pro- and anti-angiogenic proteins, either in soluble form or within EVs, depending on retinal location and the expression of the proteins involved in synthesis and release within these glial cells.

Although this review focuses on cytokines and growth factors (protein-based molecules), it is well known that a broad set of RNA molecules (e.g., miRNAs, ncRNAs, etc.) directly modulate angiogenesis in various tissues, including the retina (Biswas et al., 2021; Gandhi et al., 2024; Vishwakarma and Kaur, 2023). Furthermore, recent ophthalmological research has increasingly focused on understanding the involvement of fatty acids and lipophilic molecules in retinal angiogenesis, which is directly linked to the release of protein molecules that control this process (Elmasry et al., 2019; Fleming, 2019; Gabrielle, 2022). Understanding these processes at the molecular and cellular levels and within the context of the entire tissue will enable us to design targeted therapies aimed at key cellular players involved in the development of vasoproliferative retinopathies.

## 6 Conclusion

The pivotal involvement of Müller glial cells in retinal angiogenesis during both normal physiological conditions and pathological states cannot be understated. Through the secretion of diverse growth factors and cytokines, MGCs profoundly influence the proliferation and activity of endothelial cells, pericytes, and various retinal cell types. Unraveling the intricate molecular mechanisms underlying MGC-mediated angiogenesis holds tremendous research significance and has substantial clinical implications. Leveraging the potential of MGCs may pave the way for innovative therapeutic interventions targeting retinal vasoproliferative disorders, presenting promising avenues for future investigation and developing novel treatment strategies.
